# The Effect of Ordinary Portland Cement Substitution on the Thermal Stability of Geopolymer Concrete

**DOI:** 10.3390/ma12162501

**Published:** 2019-08-07

**Authors:** Hongen Zhang, Lang Li, Tao Long, Prabir Kumar Sarker, Xiaoshuang Shi, Gaochuang Cai, Qingyuan Wang

**Affiliations:** 1 Key Laboratory of Deep Underground Science and Engineering (Ministry of Education), School of Architecture and Environment, Sichuan University, Chengdu 610065, China; 2 Department of Civil Engineering, Curtin University, GPO Box U1987, Perth, WA 6845, Australia; 3 Failure Mechanics and Engineering Disaster Prevention and Mitigation Key Laboratory of Sichuan Province, Sichuan University, Chengdu 610207, China; 4 Laboratoire de Tribologie et de Dynamique des Systèmes (LTDS), Ecole Nationale d’Ingénieurs de Saint-Etienne (ENISE), University de Lyon, UMR 5513, 58 Rue Jean Parot, 42023 Saint-Etienne, France; 5 School of Mechanical Engineering, Chengdu University, Chengdu 610106, China

**Keywords:** elevated temperatures, residual compressive strength, mass loss ratio, geopolymer concrete, secondary geopolymerization

## Abstract

The influence of using cement on the residual properties of fly ash geopolymer concrete (FAGC) after exposure to high temperature of up to 800 °C was studied in terms of mass loss, residual compressive strength and microstructure. The mass loss was found to increase with the increase of exposure temperature, which is attributed to vaporization of water and dehydroxylation of sodium aluminosilicate hydrate (N-A-S-H) gels. The dehydroxylation of calcium silicate hydrate (C-S-H) gels and the disintegration of portlandite were responsible for higher mass loss ratio of FAGCs containing cement. The results showed that cement could increase compressive strength of FAGCs up to 200 °C, after which a significant reduction in residual strength was observed. It was found that FAGCs without cement yielded higher residual strength than the original strength after heating up to 600 °C. The observed increase of compressive strength up to 200 °C was attributed to the secondary geopolymerization which was evidenced in the scanning electronic microscopy (SEM) images.

## 1. Introduction

The continuous increasing demand of cement and the associated emission of a large quantity of carbon dioxide is a major sustainability issue for cement industry. Hence, geopolymer was created by researchers as an alternative low-emission binder to cement in concrete industry [1]. Geopolymer is an inorganic polymer produced by alkaline activation of raw materials that are affluent in silicates and aluminate [2] and geopolymer plays the binder part in geopolymer concrete. The typical raw materials used for the manufacturing of geopolymer concrete include fly ash, metakaolin and slag [3,4,5]. Since geopolymer concrete uses industrial by-products instead of energy-intensive cement, its use in construction can help improve the sustainability features of concrete industry.

Fly ash-based geopolymer concrete (FAGC) can be regarded as an emerging low-emission concrete which is made using fly ash-based geopolymer binder. In recent years, FAGC has been drawing more and more attention because of its excellent mechanical performance as well as good durability [6,7]. However, low calcium fly ash based geopolymers usually needs heat curing to accelerate setting and hardening [8], which limits its practical engineering application. Therefore, attempts have been made to produce FAGC with required properties by curing at ambient temperature [9,10,11]. It is well-known that Portland cement-based concrete develops the strength required for in-situ construction at room temperature because the hydration reaction of cement occurs at ambient temperature. Thus, several researchers succeeded to utilize a small percentage of Portland cement to improve mechanical properties and microstructure of FAGC at room temperature [12,13,14]. Nath and Sarker [12] proposed that the inclusion of an appropriate proportion of ordinary Portland cement (OPC) could promote the geopolymerization process and help geopolymer concretes obtain acceptable workability and desirable strength.

Since exposure to extreme temperatures and accidental fire have detrimental effects on properties of concrete, it is essential to evaluate the residual strength of geopolymer concrete after being exposure to elevated temperatures. This knowledge is important for assessment of the performance of concrete structures after an event of accidental fire. As a newly developing concrete, much more researches are needed to understand the effect of elevated temperature on properties of FAGC. Several past works [15,16,17,18] reported the performance of FAGCs after exposure to elevated temperatures and the results were impressive. Abdulkareem et al. [16] investigated physical and mechanical behavior of fly ash geopolymeric composites after elevated temperature exposure. The tested results showed that geopolymeric composites experienced strength at elevated temperatures. They stated that the deterioration of geopolymers was caused by the expansion of residual silicate phase. Sarker et al. [15] analyzed spalling behavior and mechanical performance of fly ash geopolymer concrete after exposure to simulated fire. They stated that geopolymer concrete presented considerably better residual properties than OPC concrete after exposure to fire. However, those works focused mainly on heat-cured specimens and only few studies were found on ambient-cured specimens, especially the ambient-cured specimens containing cement. It is important to study the behavior of ambient-cured specimens containing cement at elevated temperature because this experience will provide theoretical supports for engineering applications. Therefore, this study aimed at investigating the effect of cement on the thermal stability properties of ambient-cured FAGCs after exposure to elevated temperatures.

## 2. Materials and Methods

### 2.1. Materials

**Figure 1:**
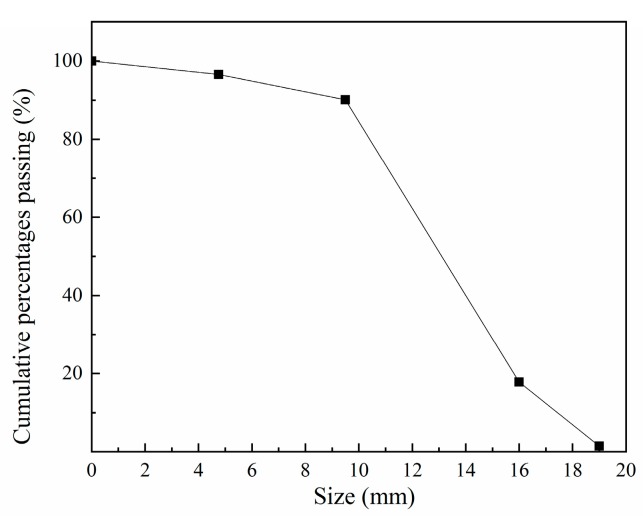
The particle size distribution of coarse aggregate.

The fine and coarse aggregates were river sand and crushed rock, respectively. The maximum size of fine and coarse aggregate were 4 mm and 22 mm, respectively. The packing densities of fine and coarse aggregate were 1342 kg/m^3^ and 1479 kg/m^3^. The apparent densities of fine and coarse aggregate were 2381 kg/m^3^ and 2632 kg/m^3^. Figure 1 shows the particle size distribution of coarse aggregate.

The sodium hydroxide (NaOH, Chengdu Kelong Chemical Reagent Factory, Chengdu, China) solution of 10 M was prepared by mixing NaOH particles of 96% purity with distilled water approximately 24 h prior to mixing FAGC. The alkaline solution was prepared by mixing sodium silicate (Na_2_SiO_3,_ Foshan Zhongfa Silicate CO.LTD, Foshan, China) solution and NaOH solution at a mass ratio of 2.5:1. The chemical compositions of Na_2_SiO_3_ solution were Na_2_O = 15.4%, SiO_2_ = 38.5% and H_2_O = 46.1% by mass and the modulus ratio (*Ms*) was 2.5.

**Figure 2:**
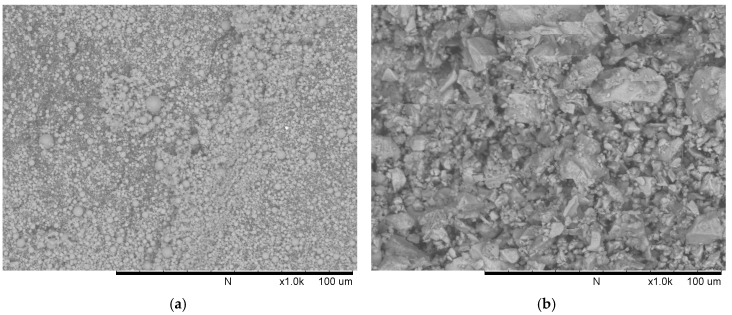
Scanning electron micrographs of: (**a**) fly ash; (**b**) cement.

Commercially available low-calcium fly ash (ASTM C618 Class F [19], Shenzhen Dot Technology Co., Ltd, Shenzhen, China) was used as the main binder material in geopolymer concrete. It can be seen from Figure 2a that the fly ash particles are of spherical shape. The Chinese 42.5R ordinary Portland cement (Sichuan Lanfeng Cement Co.,Ltd, Chengdu, China) conforming with the requirements of the national industry standard “GB 175-2007/XG1-2009” [20] was used in this study. The cement particles are of irregular shape (Figure 2b). The chemical compositions determined using X-ray fluorescence (XRF, XRF-1800, SHIMADZU, Kyoto, Japan) and loss on ignition (LOI) of fly ash and cement were presented in Table 1. Additionally, the mineral compositions of OPC are illustrated in Table 2.

### 2.2. Specimen Preparation

Three series of concrete were prepared to carry out the relevant investigation. The concrete mixture proportions are given in Table 3 and the used cement contents were 0%, 5%, and 10% by weight of the total binder (cement and fly ash).

The concrete mixtures were mixed using a laboratory pan mixer. The aggregates and binders were first mixed for about 2 min. The alkaline solution was then gradually added and the mixing was continued for about 3 min to obtain a consistent mixture. The fresh concrete was cast in 100-mm cube moulds in two layers on a vibration table. Subsequently, the specimens were covered by a plastic film and cured in a controlled condition at 20 °C and 65% relative humidity for 24 h. The specimens were then removed from the moulds and stored in a controlled condition at 20 °C in accordance with “GB/T 50081-2002” [21] until the testing age.

### 2.3. Heating Details

**Figure 3:**
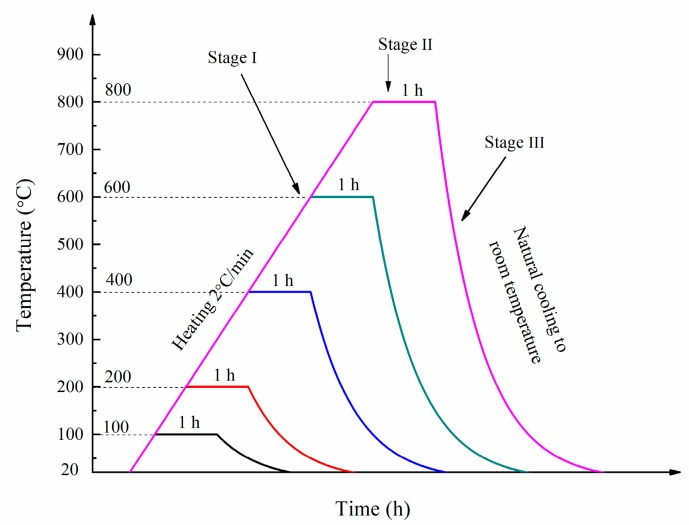
The heating regime.

A number of samples were exposed to five different temperatures, namely 100, 200, 400, 600 and 800 °C after curing for 90 days in room temperature. In stage I, the furnace temperature was increased from room temperature to the target temperature at a heating rate of 2 °C/min. In stage II, the target temperature was maintained for. In stage III, the furnace was turned off to allow the specimens to cool down to ambient temperature naturally. The heating regimes used in this study are presented in Figure 3.

### 2.4. Determination of Mass Loss by High Temperature Exposure

All samples exposed to each target temperature were also used for determination of the mass loss ratio of FAGC by Equation (1). The average mass loss ratio was found from three identical specimens.

(1) $$R=\frac{M_{b}-M_{a}}{M_{b}}\times 100\%$$

where is the percentage of mass loss, $M_{b}$ and $M_{a}$ are the mass of sample before and after exposure to the target temperature.

### 2.5. Compressive Strength Test

A 2000 kN electro-hydraulic mechanical testing machine was used to obtain the compressive strength of concrete specimens. The initial and residual compressive strength of FAGC samples were obtained with a loading rate at 0.5 MPa/s according to GB/T50081-2002 [21]. This test procedure was used to evaluate the residual compressive strength value of FAGC after exposure to elevated temperature. The strength of three samples was obtained to determine the average compressive strength values according to GB/T50081-2002 [21].

### 2.6. Scanning Electron Microscopic Images

Scanning electron microscopy (SEM) was conducted with TM3000 (HITACHI, Kyoto, Japan) instrument to investigate the microstructural deterioration of the FAGC samples because of exposure to elevated temperatures. The fragment of sample without coarse aggregate was selected to perform SEM.

### 2.7. Thermogravimetric Analysis (TGA)

The aggregates were weeded out when preparing the samples for the thermogravimetric analysis (TGA) test. Fragments of FAGC specimens unexposed to elevated temperatures were powdered to conduct the TGA test through METTLER TOLEDO TGA/DSC2/1600 (METTLER TOLEDO, Zurich, Switzerland) instrument in alumina crucibles in the nitrogen environment (N_2_ flowing at 50 mL/min), and then elevated to about 800 °C with a constant heating rate of 10 °C/min in the same gas environment. One sample for each group was prepared to conduct the TGA test.

## 3. Results and Discussions

### 3.1. Mass Change by High Temperature Exposure

**Figure 4:**
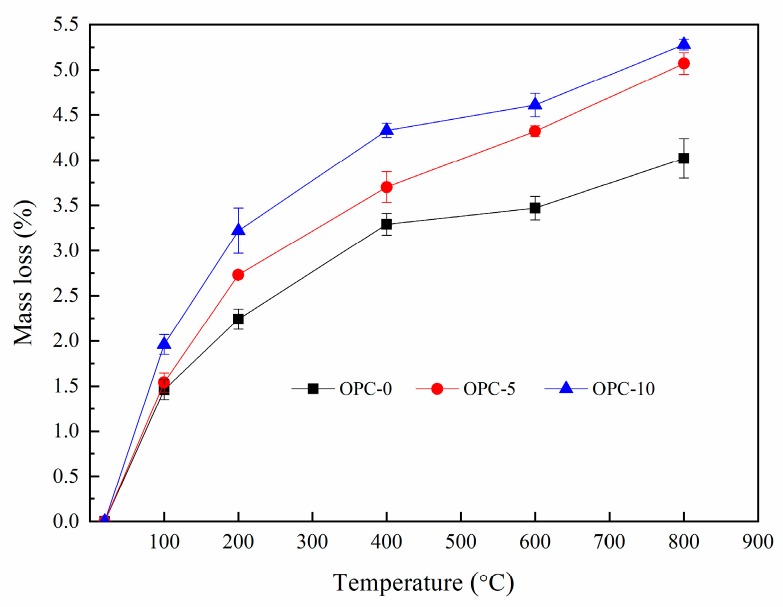
The percentage of mass loss at elevated temperatures.

The percentage of mass loss after exposure to elevated temperatures were presented in Figure 4. It can be seen from Figure 4 that the percentage of mass loss increased with the increases of temperature and the cement content. It is observed from Figure 4 that the rate of mass change slowed down after 200 °C, indicating that the mass loss mainly occurred before 200 °C. The mass loss occurred within this temperature range accounted for more than 50% of the total mass loss.

**Figure 5:**
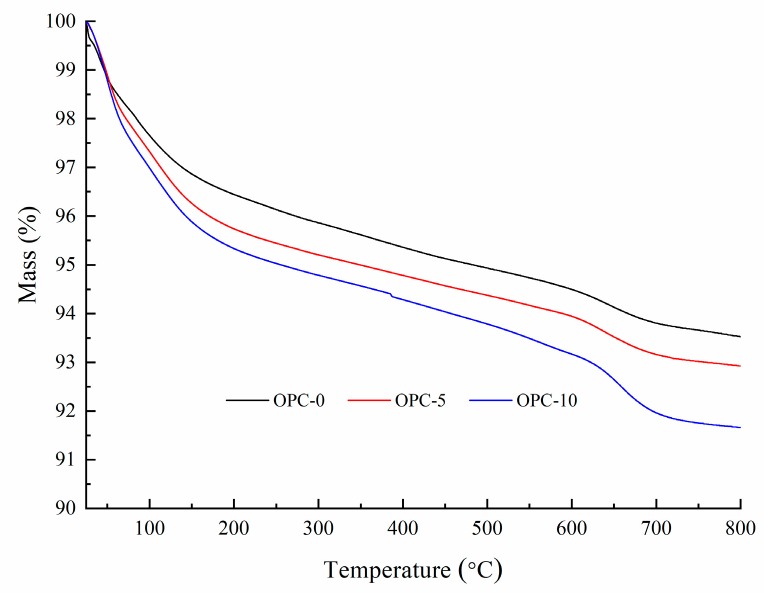
The TGA curves of low-calcium FAGC samples.

**Figure 6:**
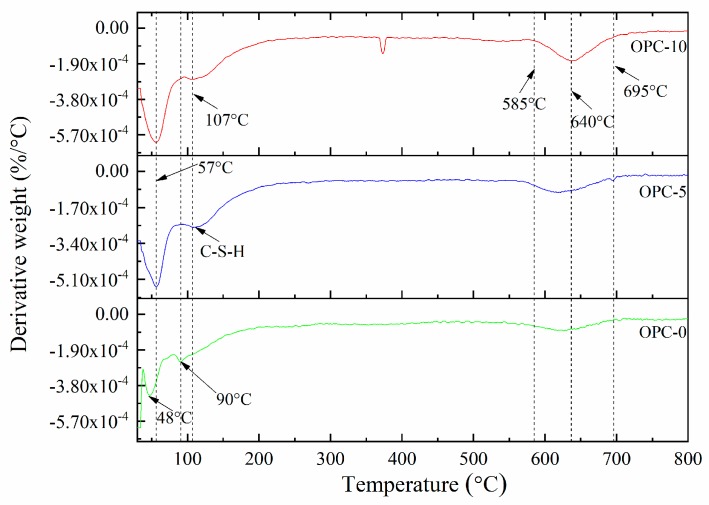
The DTG curves of low-calcium FAGC samples.

The thermogravimetric analysis (TGA) and derivative thermogravimetry (DTG) of geopolymer concrete are presented in Figure 5 and Figure 6, respectively. It can be observed in Figure 5 that the first mass loss peak of OPC-0 occurred at about 48 °C and those of OPC-5 and OPC-10 occurred at about 57 °C, which was believed to be the evaporation of free water existing in concrete. Therefore, the mass loss occurred before 100 °C is attributed to the loss of free water. The second peak of OPC-0 occurred at about 97 °C and those of OPC-5 and OPC-10 occurred at 107 °C in Figure 5 could be attributed to the loss of chemically combined water in the N-A-S-H gel [22,23,24] and portlandite, C-S-H and ettringite [25,26], which could be responsible for the mass loss occurred between 100 °C and 200 °C. The inclusion of OPC increased the calcium content, resulting in an increase in portlandite, C-S-H and ettringite in the FAGC samples. Therefore, the percentage of mass loss increased with the OPC content. The heat curing method could accelerate the rate of geopolymerization and improve the amounts of reaction products. Since the cement hydration process is an exothermic action [27], the cement hydration heat could promote the geopolymerization and increase the quantity of N-A-S-H gel. This could be regarded as another reason to explain why the percentage of mass loss increased with the increase of OPC content.

The mass loss between 200 and 400 °C is attributed to continuous dehydroxylation of N-A-S-H gel [28] and C-S-H [25,29]. This is consistent with the information presented in Figure 5 that the mass loss rate of OPC-0 was constant between 200 °C to 400 °C. At the same time, the loss of interlayer water in pores of N-A-S-H as well as to the first stage of dehydroxylation makes contributions to the mass loss occurred within this temperature range. It is similar to the explanation proposed by several authors [30,31,32] when they investigated cement-based concrete. Additionally, the mass loss of OPC-5 and OPC-10 occurred within this temperature range is also resulted from the dehydration of C-S-H. As can be seen from the DTG curve of OPC-0 (Figure 6), the mass loss occurred in the range of about 585 °C to 695 °C is associated with the dehydroxylation of OH groups, such as sodium-based geopolymers. But the dehydroxylation of OH groups made a very little contribution to the mass loss of OPC-5 and OPC-10 samples within this temperature range. The mass loss of OPC-5 and OPC-10 was mainly induced by the decomposition of Ca(OH)_2_ in this temperature range. The DTG diagram implied that much more Ca(OH)_2_ was produced in FAGC containing 10% cement. Finally, the mass loss rate stabilized again after 695 °C, which can be seen from Figure 6.

### 3.2. Compressive Strength after Exposure to Elevated Temperatures

**Figure 7:**
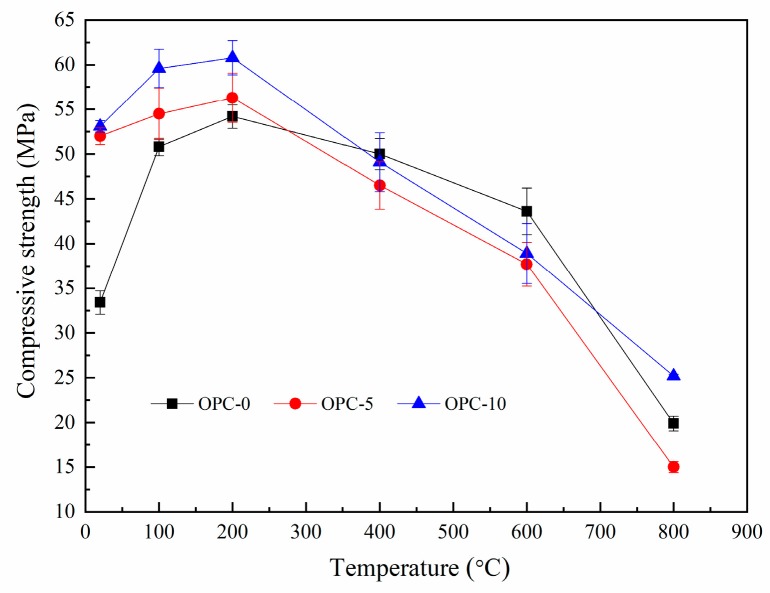
Residual compressive strength of low-calcium FAGC.

The compressive strengths of FAGC before and after exposure to target temperatures are illustrated in Figure 7. It can be seen from Figure 7 that the compressive strength of FAGCs without cement increased up to 200 °C and declined during the temperature range of 200–800 °C. The trend of this change is consistent with that of heat-cured geopolymer concrete samples [28,33]. The improvement in compressive strength up to 200 °C resulted from the secondary geopolymerization occurring during the heating process, which was one hypothesis promoted by other authors [34]. In general, FAGC should be cured at the temperature ranging from 50 °C to higher temperature to achieve more complete geopolymerization. In view of this fact, some fly ash particles and silicate phases would be left unreacted if geopolymer concrete was cured at room temperature. Therefore, the results could provide a strong experimental support for the above hypothesis. Türkmen et al. [35] also believed that the beneficial effect of thermal drying was helpful for the increase of compressive strength until 200 °C. Up to 200 °C, the enhancement in compressive strength of FAGCs containing cement also resulted from the following two primary reasons. Firstly, the product of cement hydration reaction could improve compressive strength. Secondly, the filler effect of any unreacted cement particles that filled the interstitial spaces existing in the concrete structure resulted in the enhancement of compressive strength [33].

The significant decrease of compressive strength was largely associated with the following reasons. Firstly, the compressive strength reduction was related to the evaporation of chemically combined water, which could be observed from Figure 6. This is because that this typical water plays as an indispensable part of the structure of geopolymer gels. In addition, there was a mass loss rate peak between 585 and 695 °C (Figure 6), indicating the dehydroxylation of N-A-S-H gel and other hydrates (C-S-H and ettringite) gel, which was also responsible for the reduction of compressive strength. Being exposure to 800 °C brought about a dramatic decline in compressive strength for all groups, which can be mainly ascribed to the serious microstructural deterioration including large cracks produced by excessive vapor pressure and the difference in thermal strain of geopolymer and aggregates. The strength loss caused by the microstructural deterioration is further discussed in Section 3.3. Additionally, the cement hydration product dehydrated at this temperature and led to a decline in compressive strength [35].

The change trend of compressive strength of FAGC at each target temperature can also be obtained from Figure 7. It was easy to find that the improvement in compressive strength mainly occurred at 100 °C and the loss of compressive strength largely took place at 800 °C. At 100 °C, the percentage of compressive strength enhancement of OPC-0 was about 52.1%, which was much higher than that of OPC-5 (4.8%) and OPC-10 (12.2%), indicating that secondary geopolymerization made more contributions to the compressive strength improvement of OPC-0 samples. Research [36] reported that water played a vital part in providing the essential liquid environment for dissolution of fly ash particles, hydrolysis and condensation reactions as geopolymerization process continues which could be obtained by Equations (2) and (3) [37]. There was much more free water in the geopolymer concrete without cement than specimens containing cement. It was because that some free water was consumed by the cement hydration in specimens containing cement. Consequently, the process of the destruction of fly ash particles and hydrolysis of existing Al^3+^ and Si^4+^ happened in the FAGCs without cement during geopolymer synthesis (referring to Equation (2)) was promoted [36], which led to relatively much more complete secondary geopolymerization up to 100 °C. Therefore, geopolymer concrete without cement experienced much more significant secondary geopolymerization, resulting in greater improvement of compressive strength. At 800 °C, the reduction in compressive strength of OPC-0, OPC-5 and OPC-10 were 54.4%, 60.2% and 35.2% respectively, indicating OPC-5 samples experienced the most severe compressive strength deterioration.

(2) $${\left({\mathrm{Si}}_{2}O_{5},{\mathrm{Al}}_{2}O_{2}\right)}_{n}+{\mathrm{nSiO}}_{2}+4{\mathrm{nH}}_{2}O+{\mathrm{nOH}}^{-}\;\to \;{n(\mathrm{OH})}_{3}-\mathrm{Si}-O-\underset{{\left(\mathrm{OH}\right)}_{2}}{\underset{|}{{\mathrm{Al}}^{-}}}{-O-\mathrm{Si}-(\mathrm{OH})}_{3}$$

(3) $$\begin{array}{l} {n(\mathrm{OH})}_{3}-\mathrm{Si}-O-\underset{{\left(\mathrm{OH}\right)}_{2}}{\underset{|}{{\mathrm{Al}}^{-}}}{-O-\mathrm{Si}-(\mathrm{OH})}_{3}+\mathrm{NaOH}\;\mathrm{or}\;\mathrm{KOH}\;\to \;{(\mathrm{Na}}^{+}{,K}^{+})-\underset{\mathrm{Geopolymeric}\;\mathrm{structure}}{\left(-\underset{\underset{|}{O}}{\overset{|}{\underset{|}{\mathrm{Si}}}}-O-\underset{\underset{|}{O}}{\overset{|}{\underset{|}{{\mathrm{Al}}^{-}}}}-O-\underset{|}{\underset{O}{\overset{|}{\underset{|}{\mathrm{Si}}}}}-O-\right)}+{4\mathrm{nH}}_{2}O \end{array}$$

**Figure 8:**
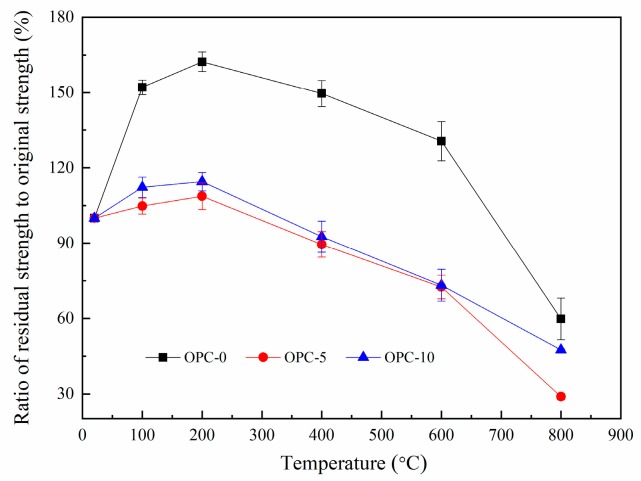
Ratio of residual compressive strength to original compressive strength.

The ratio of residual compressive strength to original compressive strength is illustrated in Figure 8. The ratios for OPC-0, OPC-5 and OPC-10 are about 167%, 103%, and 114% respectively at 200 °C and the values are about 60%, 29% and 48% respectively at 800 °C. These residual strengths are higher than those usually found for cement-based concrete [38] and heat-cured fly ash-based geopolymer concrete [18].

### 3.3. Microstructure Investigation

**Figure 9:**
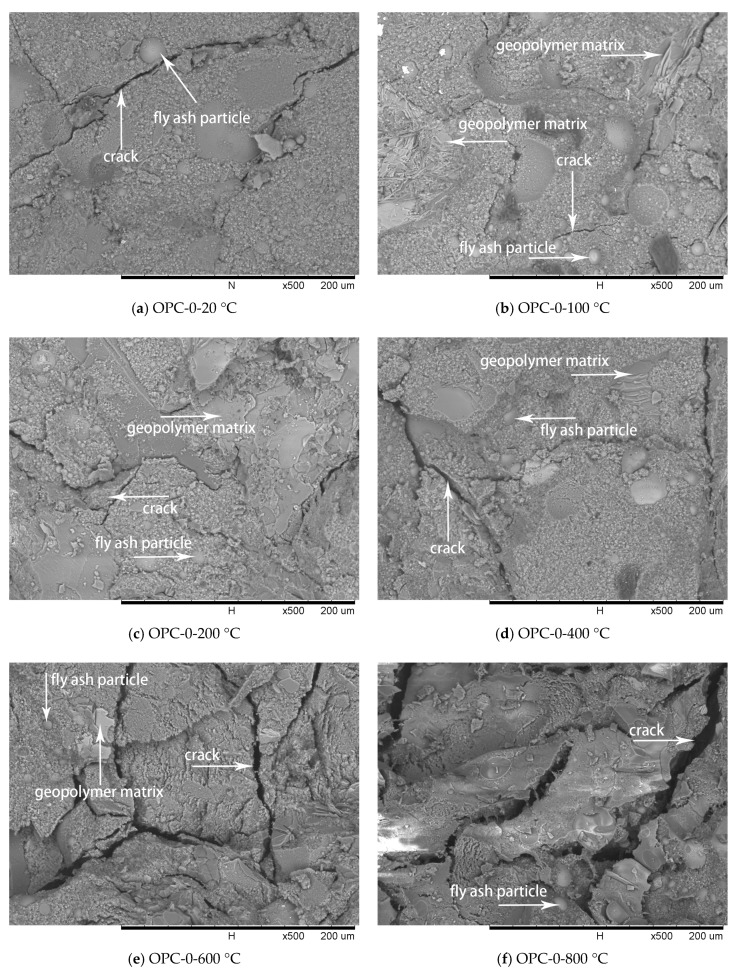
SEM micrographs of OPC-0 series at different temperatures.

**Figure 10:**
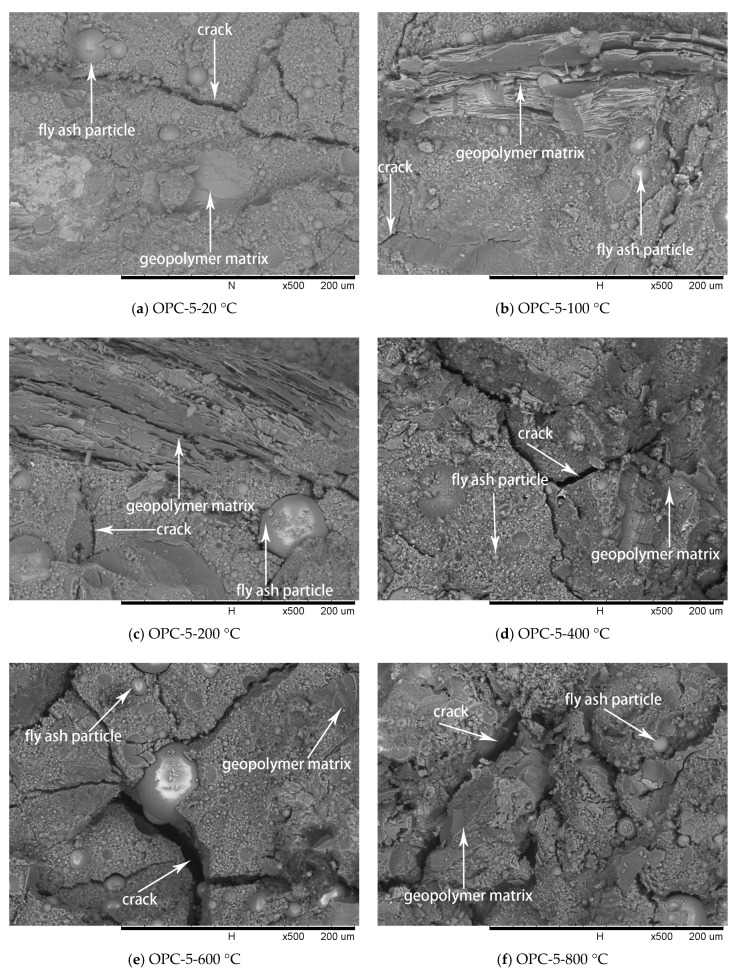
SEM micrographs of OPC-5 series at different temperatures.

**Figure 11:**
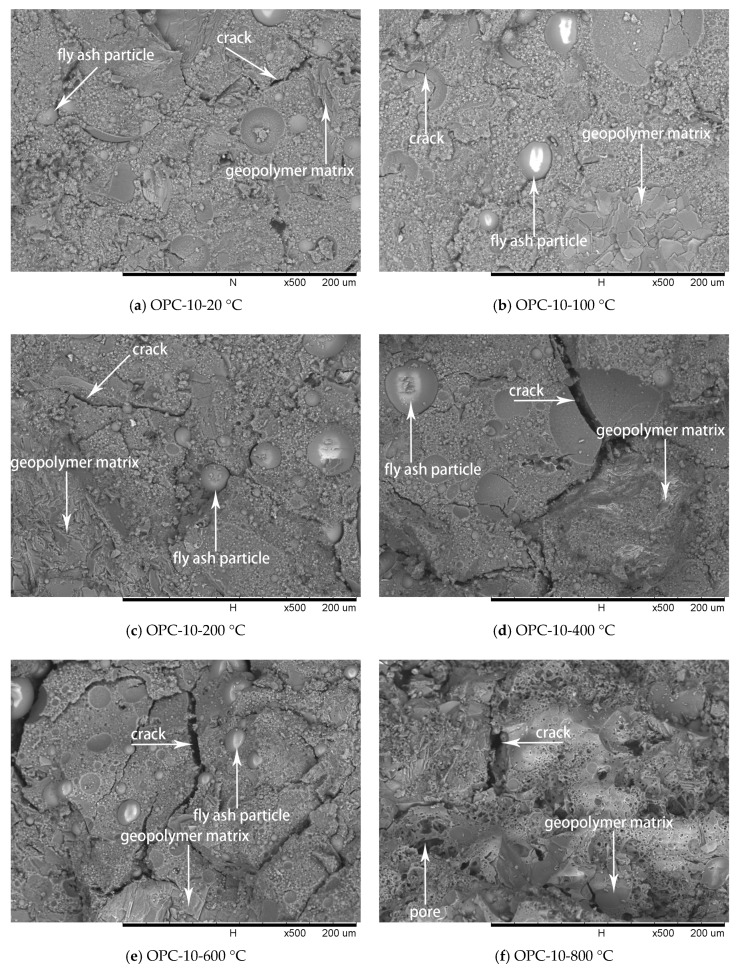
SEM micrographs of OPC-10 series at different temperatures.

SEM analysis was used to evaluate the characteristic of FAGC microstructure at different temperatures and the results are illustrated in Figure 9, Figure 10 and Figure 11.

Comparing with Figure 2a, the spherical particles in Figure 9 are believed to be unreacted fly ash particles. The residual fly ash particles would provide essential materials for the potential secondary geopolymerization. The cracks of geopolymer matrix after exposures to 100 °C and 200 °C, as shown in Figure 9b,c, are less wide than those of the matrix at 20 °C, as shown in Figure 9a. The reduction of crack width in the matrix after the exposures of 100 °C and 200 °C is attributed to the increase of reaction products by the secondary geopolymerization that occurred under exposure temperatures. Therefore, the growth in compressive strength of FAGCs up to 200 °C is attributed to secondary geopolymerization, which improved the microstructure of concrete. The quantity of cracks increased and the width of cracks widened in the temperature range of 400 °C to 800 °C that deteriorated the internal structure of FAGC and eventually reduced the compressive strength. Exposure to 800 °C resulted in the highest deterioration of microstructure as illustrated in Figure 9f. Thus, the FAGCs had the lowest residual compressive strength (Figure 7) after being subjected to 800 °C. The difference in thermal expansions of geopolymer matrix and aggregates also contributed to the deterioration of microstructure and expansion of cracks. Additionally, the vapor pressure caused by the vaporization of free water as well as chemically combined water resulted in further development of cracks. The swelling of residual silicate were also responsible for the degradations of microstructure and compressive strength deterioration [16].

The SEM images of OPC-5 and OPC-10 before and after exposure to elevated temperatures are presented in Figure 10 and Figure 11. It is observed from Figure 10a and Figure 11a that use of cement could improve the microstructure, resulting in higher initial compressive strength of FAGCs. Unreacted fly ash particles were also observed in FAGCs containing cement, therefore, the strength enhancement in OPC-5 and OPC-10 series up to 200 °C was also due to secondary geopolymerization. Being consistent with the microstructure of OPC-0 samples, the microstructures of OPC-5 and OPC-10 concrete samples also suffered from severe deterioration in microstructure within the temperature range of 400 °C to 800 °C. It was observed that OPC-5 and OPC-10 samples suffered more severe microstructure deterioration than OPC-0 samples. Therefore, the residual compressive strength of FAGCs containing cement was lower than the samples of OPC-0 series overall during the temperature range of 400 °C to 800 °C. It is important to understand why cement would have harmful effect on the elevated temperature resistance of FAGC after exposure to 400 °C. Firstly, Abdulkareem et al. [16] showed that the elevated temperature resistance of geopolymer concrete could be significantly weakened by the densification and swelling process of residual silicate phases within the temperature range of 600–800 °C. Cement could help FAGC to produce a relatively highly dense microstructure at ambient temperature, which could be observed from Figure 9a, Figure 10a and Figure 11a. Therefore, OPC-5 and OPC-10 would suffer more microstructural deterioration according to the observation of Abdulkareem et al. [16]. Secondly, the introduction of cement tended to increase the Ca/Si ratio and quantities of C-S-H gel due to the high CaO content in cement. The promotion in the Ca/Si ratio value and the amounts of C-S-H weakened the elevated temperature resistance of FAGC [39]. The statement was also supported by the results of OPC-10 up to 600 °C.

No visible particles are observed in Figure 11f and OPC-10 samples experienced larger mass loss between 600 and 800 °C (Figure 5 and Figure 6), which implied that the unreacted fly ash particles have fused and melted within this temperature range. Rickard et al. [40] argued that the increase of strength at temperature higher than 800 °C was ascribed to the sintering and melting. However, the sintering and melting seemed to have occurred at temperature higher than 600 °C in OPC-10 series (Figure 11f). Therefore, OPC-10 specimens performed higher residual compressive strength than OPC-0 specimens at 800 °C (Figure 7). It can be observed from Table 1 and Table 2 that the inclusion of cement could increase the Ca/Si ratio in FAGC specimens, indicating the increasing Ca/Si ratio might facilitate the sintering and melting appeared at lower temperatures.

## 4. Conclusions

This paper presented behavior of ambient-cured low-calcium FAGCs containing different, ordinary Portland cement dosages after exposure to elevated temperatures. The following conclusions could be derived from the experimental results:

1. The mass loss occurred at temperatures up to 200 °C accounted for more than 50% of the entire mass loss, which mainly resulted from the evaporation of free water and chemically bound water. The mass loss ratio increased with the cement content, which was caused by the continuous dehydroxylation of C-S-H gels as well as the disintegration of Ca(OH)_2_ at elevated temperatures.
2. All the FAGCs performed higher compressive strength than their original compressive strength up to 200 °C, which was due to the secondary geopolymerization resulting from unreacted fly ash and alkaline solution. FAGCs without cement experienced higher strength enhancement than those containing cement. More impressive, FAGCs without cement retained higher compressive strength than its original compressive strength until 600 °C.
3. The SEM images demonstrated that the compressive strength enhancement resulted from secondary geopolymerization that produced further geopolymer gels and improved the concrete microstructure. SEM, TGA and DTG results revealed that the compressive strength loss occurred at temperatures higher than 200 °C was mainly caused by the microstructure deterioration and the continuous dehydroxylation of N-A-S-H gel.
4. Compressive strength of FAGCs increased with the cement dosages up to 200 °C because of the existence of C-S-H gels and filler effect of cement. According to the SEM results, the fact that cement was beneficial for improving microstructure of FAGCs caused the compressive strength enhancement.
5. The inclusion of cement increased the Ca/Si ratio, which resulted in an increase in the quantity of calcium compounds with strong thermal expansion properties. Therefore, FAGCs containing cement suffered from more severe strength loss and microstructure deterioration than FAGCs without cement at temperatures higher than 200 °C.

## Figures and Tables

**Table 1:** Chemical composition of fly ash and cement.

Chemical Composition	SiO_2_	Al_2_O_3_	CaO	Fe_2_O_3_	MgO	K_2_O	SO_3_	TiO_2_	Na_2_O	LOI ^†^
Fly Ash (%)	49.05	26.40	5.2	4.64	3.72	4.85	2.00	1.16	0.8	2.83
Cement (%)	17.78	2.49	63.67	2.5	3.09	0.46	4.77	0.80	0	4.53

^†^ Loss on ignition.

**Table 2:** The mineral compositions of cement.

Mineral Composition	Ca_3_SiO_5_	Ca_2_SiO_4_	Ca_2_Fe_1.40_ Al_0.60_O_5_	CaSO_4_	CaCO_3_
SemiQuant (%)	44.55	38.61	3.98	6.92	5.94

**Table 3:** Mixture design of the FAGC.

Mixture Design of the FAGC (kg/m^3^)					
Mixes	Coarse Aggregate	Sand	Fly Ash	OPC	Alkaline Solution
OPC-0	1172	539	459	0	200
OPC-5	1172	539	436.05	22.95	200
OPC-10	1172	539	413.10	45.9	200
